# Risk assessment for indeterminate pulmonary nodules using a novel, plasma-protein based biomarker assay

**DOI:** 10.15761/brcp.1000173

**Published:** 2018-10-29

**Authors:** Neil N Trivedi, Mehrdad Arjomandi, James K Brown, Tess Rubenstein, Abigail D. Rostykus, Stephanie Esposito, Eden Axler, Mike Beggs, Heng Yu, Luis Carbonell, Alice Juang, Sandy Kamer, Bhavin Patel, Shan Wang, Amanda L Fish, Zaid Haddad, Alan HB Wu

**Affiliations:** 1 San Francisco Veterans Affairs Medical Center, 4150 Clement St, San Francisco, CA, USA; 2 The University of Pennsylvania, Philadelphia, USA; 3 The University of Michigan, 500 S State St, Ann Arbor, MI, USA; 4 MagArray Inc, Milpitas, CA, USA; 5 Vancouver, BC, Canada; 6 University of California, San Francisco, USA

**Keywords:** biomarkers, diagnosis, lung cancer, pulmonary nodules, risk models

## Abstract

**Background:**

The increase in lung cancer screening is intensifying the need for a noninvasive test to characterize the many indeterminate pulmonary nodules (IPN) discovered. Correctly identifying non-cancerous nodules is needed to reduce overdiagnosis and overtreatment. Alternatively, early identification of malignant nodules may represent a potentially curable form of lung cancer.

**Objective:**

To develop and validate a plasma-based multiplexed protein assay for classifying IPN by discriminating between those with a lung cancer diagnosis established pathologically and those found to be clinically and radiographically stable for at least one year.

**Methods:**

Using a novel technology, we developed assays for plasma proteins associated with lung cancer into a panel for characterizing the risk that an IPN found on chest imaging is malignant. The assay panel was evaluated with a cohort of 277 samples, all from current smokers with an IPN 4–30 mm. Subjects were divided into training and test sets to identify a Support Vector Machine (SVM) model for risk classification containing those proteins and clinical factors that added discriminatory information to the Veteran’s Affairs (VA) Clinical Factors Model. The algorithm was then evaluated in an independent validation cohort.

**Results:**

Among the 97 validation study subjects, 68 were grouped as having intermediate risk by the VA model of which the SVM model correctly identified 44 (65%) of these intermediate-risk samples as low (n=16) or high risk (n=28). The SVM model negative predictive value (NPV) was 94% and its sensitivity was 94%.

**Conclusion:**

The performance of the novel plasma protein biomarker assay supports its use as a noninvasive risk assessment aid for characterizing IPN. The high NPV of the SVM model suggests its application as a rule-out test to increase the confidence of providers to avoid aggressive interventions for their patients for whom the VA model result is an inconclusive, intermediate risk.

## Introduction

Lung cancer is the third most common cancer and the leading cause of cancer death in the United States [1]. The most important risk factor for lung cancer is smoking, which results in approximately 85% of all US lung cancer cases [2]. Although the prevalence of smoking has decreased, approximately 1 of every 6 US adults is a current smoker [3]. The incidence of lung cancer increases with age and occurs most commonly in persons 55 years or older [4].

Lung cancer has a poor prognosis, and nearly 90% of persons with lung cancer die of the disease. Yet, when detected at an early-stage, non-small cell lung cancer (NSCLC) has much a better prognosis and can be successfully treated with surgical resection [4].

The National Lung Cancer Screening Trial showed that low-dose Computed Tomography (CT) screening results in a 20% relative mortality reduction in high risk individuals [5]. The mortality reduction, however, was accompanied by a high rate (~96%) of false-positive CT findings, which in turn has generated concern for the overuse of invasive diagnostic procedures [6]. CT identifies several million indeterminate pulmonary nodules annually, and even though most of these nodules are benign, many patients undergo unnecessary procedures. It is estimated that 350,000 bronchoscopies are performed per year in the US, where benign disease is identified in 40% of the patients; additionally, more than 9% of bronchoscopy patients experience complications from that procedure including bleeding, pneumothorax and death [7]. Moreover, 102,000 surgeries occur for benign disease, resulting in 2,052 preventable deaths annually [8]. Consequently, there is a high unmet need for a noninvasive clinical test that can discriminate between benign and malignant nodules [9,10].

Limitations of CT have spurred research on novel plasma and tissue biomarkers to aid in correctly identifying the actual risk of malignancy when the nodule appearance and clinical factors result in an inconclusive intermediate risk for lung cancer as calculated by the VA Model [9]. Current technologies have not resulted in diagnostic tests sufficiently reliable or convenient to apply to clinical practice for early detection of aggressive lung cancer [8,10,11]. Consequently, there is a need for new biomarkers and an enabling technology that can identify lung cancer at an early state of disease progression while limiting the number of false positives. This is especially true when the probability of malignancy is in the intermediate range (as calculated, for example by the VA model) and the pulmonary nodule is indeterminate as defined by a small, focal opacity in the lung measuring up to 30 mm that does not have features strongly suggestive of a benign etiology [12].

An ideal set of biomarkers would provide a signal to identify a malignant nodule whether the subject is a current, former, or never smoker. However, smoking induces significant genetic alterations in the lungs, and consequently the cellular biochemistry of a current smoker is different from that of a former smoker, and even more dissimilar to a never smoker [13]. Our development efforts focused on current smokers as the group of patients who are the most likely to be at high risk for developing lung cancer. When compared to former or never smokers, current smokers had a more consistent biomarker profile and thus a reliable set of informative biomarkers might more likely be found. With over 15% (17.8 million) of U.S. adults identified as current cigarette smokers, a significant population would be served by a noninvasive test that can more accurately discern benign from malignant nodules in this high-risk cohort of patients [3].

## Materials and methods

### Laboratory-developed test: Novel multiplexed plasma protein assay development

The discovery that patients with cancer produce detectable proteins associated with their tumor progression suggests that these biomarkers could have diagnostic and prognostic value [10]. State-of-the-art genomic and proteomic information was used to identify biomarker candidates for which sensitive and specific assays could be developed to measure subtle changes in circulating levels associated with the presence of lung cancer. Building upon these findings, we initially developed multiplexed panels of assays for seven plasma protein biological markers known to be associated with the presence of lung cancer [11]. We employed these customized assays in early discovery work to measure the biomarker levels in a cohort of subjects who were enrolled in an observational study of PET-CT imaging for lung cancer. The subjects were from three medical centers: Stanford University Clinic, California Pacific Medical Center, and Palo Alto Veterans Affairs Hospital and included individuals identified as current, former, and never smokers.

**Figure 1.**
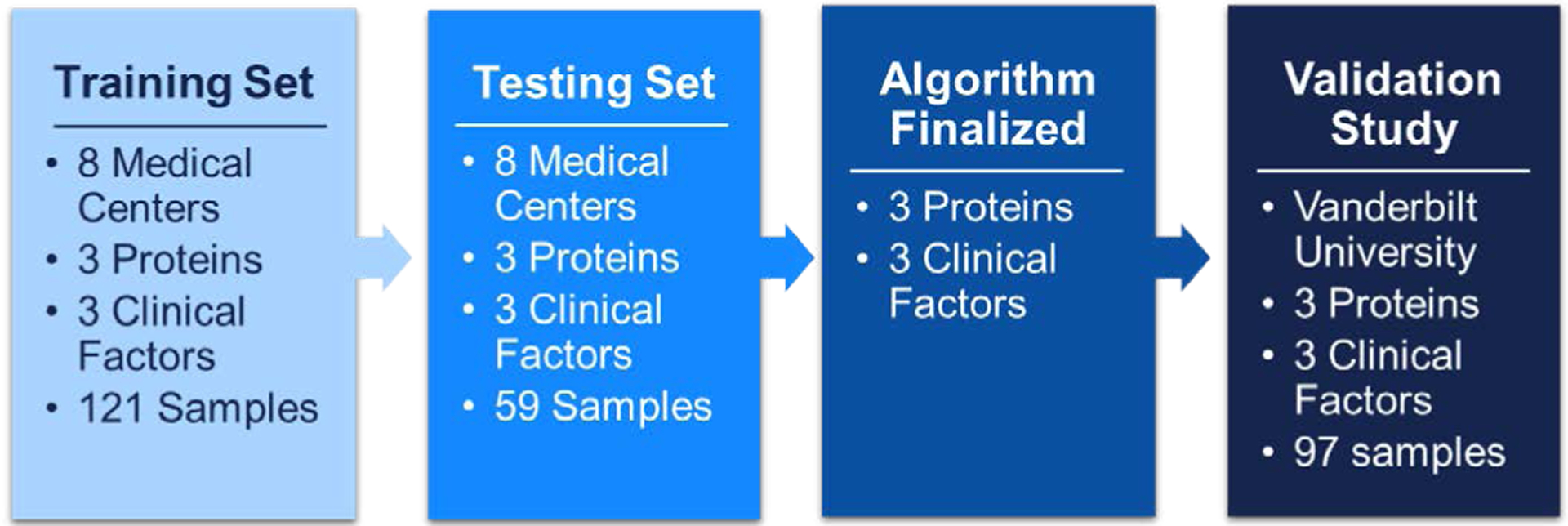
Diagram of model development process and subject cohort designations

Three proteins epidermal growth factor receptor (EGFR), prosurfactant protein B (ProSB), and tissue inhibitor of metalloproteinases 1 (TIMP1) were subsequently identified as the best at discriminating benign and malignant nodules in the subset of current smokers. We then optimized those 3 protein assays and developed a risk assessment algorithm that we trained in a more diverse set of current smokers from multiple cohorts (Figure 1).

### The MagArray technology

The novel MagArray magnetic nanosensor technology and instruments enable the sensitive detection of biomolecules in a multiplexed format that requires no optics or microfluidics, while providing a simultaneous real-time readout of up to 640 analyte specific sensors [14]. This technology was used to develop a multiplexed immunoassay test for circulating proteins, that required only 20 μL of a plasma sample to detect pg/mL analyte levels.

The three protein assays comprising the lung nodule characterization test show acceptable analytical performance demonstrating the necessary sensitivity, precision and reproducibility for use in a commercial clinical laboratory to calculate the probability of malignancy for indeterminate lung nodules found on CT scans [15].

## Research design

### Model development study population

After the development and optimization of the three selected plasma protein assays, a larger set of retrospective human plasma samples and associated clinical data were obtained from 8 geographically diverse centers including Stanford University Clinic, California Pacific Medical Center, and Palo Alto Veterans Affairs Hospital, the San Francisco Veterans Affairs Medical Center, University of Pennsylvania, and the Lung Cancer Biospecimen Resource Network (Medical University of South Carolina, University of Virginia, and Washington University at St. Louis). These specimens were used in our protein-biomarker based algorithm training and testing sets.

Frozen EDTA plasma samples were shipped to a central laboratory and kept frozen (−80°C) until processed for testing. The processing included thawing the samples and then preparing 100 μL aliquots that were refrozen to allow all testing and retesting to occur with the same number of 2 freeze-thaw cycles. Studies with freshly collected, never-frozen samples demonstrated analyte stability for up to 4 days at 2–8°C before freezing at-80°C, and up to 4 freeze-thaw cycles with no significant change in the measured analyte values [15].

No subjects were compensated. All sample collections fully complied with applicable laws, regulations and institutional polices that provide protections for human subjects. The plasma samples were used only for the research purposes specified in the original application and in accordance with the conditions and IRB stipulations specified by the centers from which the samples were sourced. Test results were not provided to the clinical sites for patient care, and the laboratory technicians who performed the biomarker tests were blinded to the subject characteristics. The protein assay development studies were all performed in accordance with the Standards for Reporting of Diagnostic Accuracy criteria [16].

All samples used in the training and test sets were from current smokers 25–85 years old with indeterminate lung nodules measuring 4 to 30 mm in diameter as indicated on the clinical data record. The training set consisted of 121 samples (2/3 of the total cohort) randomly selected from the subjects with a malignant lung nodule diagnosis and from those with benign disease. The remaining 59 samples (1/3 of the total cohort) were assigned to the test set. The prevalence of disease was 64% in the training set and 59% in the test set. Both cohorts had similar nodule size distributions and other clinical characteristics as summarized in Table 1.

The training and test sets of subject samples were measured with the protein 3-plex assay panel. The protein concentrations obtained from the training subset were used to develop an algorithm that discriminated between patients with benign and malignant pulmonary nodules (as described in the statistical analysis section). The final algorithm incorporated the three plasma proteins along with three clinical factors subject age, sex, and nodule diameter.

### Validation study population

Retrospective plasma samples and annotated clinical information for 97 pathologically or scan confirmed IPN were sourced from Vanderbilt University Medical Center. Eligible participants included current smokers with an IPN of 4 to 30 mm. Subjects with metastatic disease, or previously diagnosed lung cancer were excluded from the study. Of the 97 subjects, 49 were diagnosed with a malignant nodule and 48 had benign disease for a prevalence rate of 51%. Almost all (98%) subjects had early stage disease as defined by Lung Cancer Stage I or II (Table 2).

### Algorithm development

First, a VA Model pretest probability of malignancy was calculated for every sample. The primary objective then was to identify and validate the accuracy of the algorithm, which was based on the plasma levels of 3 proteins and 3 clinical factors, to predict benign disease versus lung cancer in current smokers 25–85 years old with indeterminate nodules (4 to 30 mm in diameter). The algorithm outcome was compared to the result calculated by the VA model which employed four independent predictors of malignant pulmonary nodules: smoking status, age, nodule size, and years since quitting smoking.

These studies fully complied with the recommendations of the 2012 report from the Institute of Medicine Committee on Omics-Based Predictive Tests [17]. Results of the studies were analyzed by an independent biostatistician.

### Variable transformation and selection

Nodule size was base 10 log transformed after adding 1 to avoid 0 values, protein biomarker concentrations were natural log transformed, subject sex was treated as a binary variable, and age was divided by 10. Both protein and clinical variables were ranked by importance using a random forest method to calculate the mean decrease in the Gini Impurity measurement.

### Algorithm development

A support vector machine (SVM) learning algorithm was used to assemble the selected features into a multidimensional classifier model with an SVM linear kernel as the starting point. A final selection step was used to optimize the feature set on the classification algorithm using a tuning function with 10-fold cross validation to optimize the model cost and gamma parameters to 2.1 and 0.5, respectively. The final SVM model produces a score between 0 and 1 indicating the risk that the nodule is malignant.

The performance of the SVM model was compared to those of the previously published VA Model which was calculated as described [9].

### Statistical analysis

Statistical analyses were performed in R v3.4.4 and all tests were two-sided using a 5% significance level. Feature ranking was performed using RandomForest v4.6–12 and model identification and tuning used e1071 v1.6–8. Optimal Cut points were developed using OptimalCutpoints v1.1–3. The performance of the algorithm was evaluated using area under ROC curves (AUC) with pROC v1.10.0 [18], confusion matrices with caret v6.0–78, and discrimination boxplots using base R and ggplot v2.21. Base R stats and Microsoft Excel v2016 were used to calculate the cohort demographic summaries, sensitivity, specificity, negative predictive value (NPV), and positive predictive value (PPV).

## Results

### Biomarker distributions in the training, testing, and validation sets

**Figure 2.**
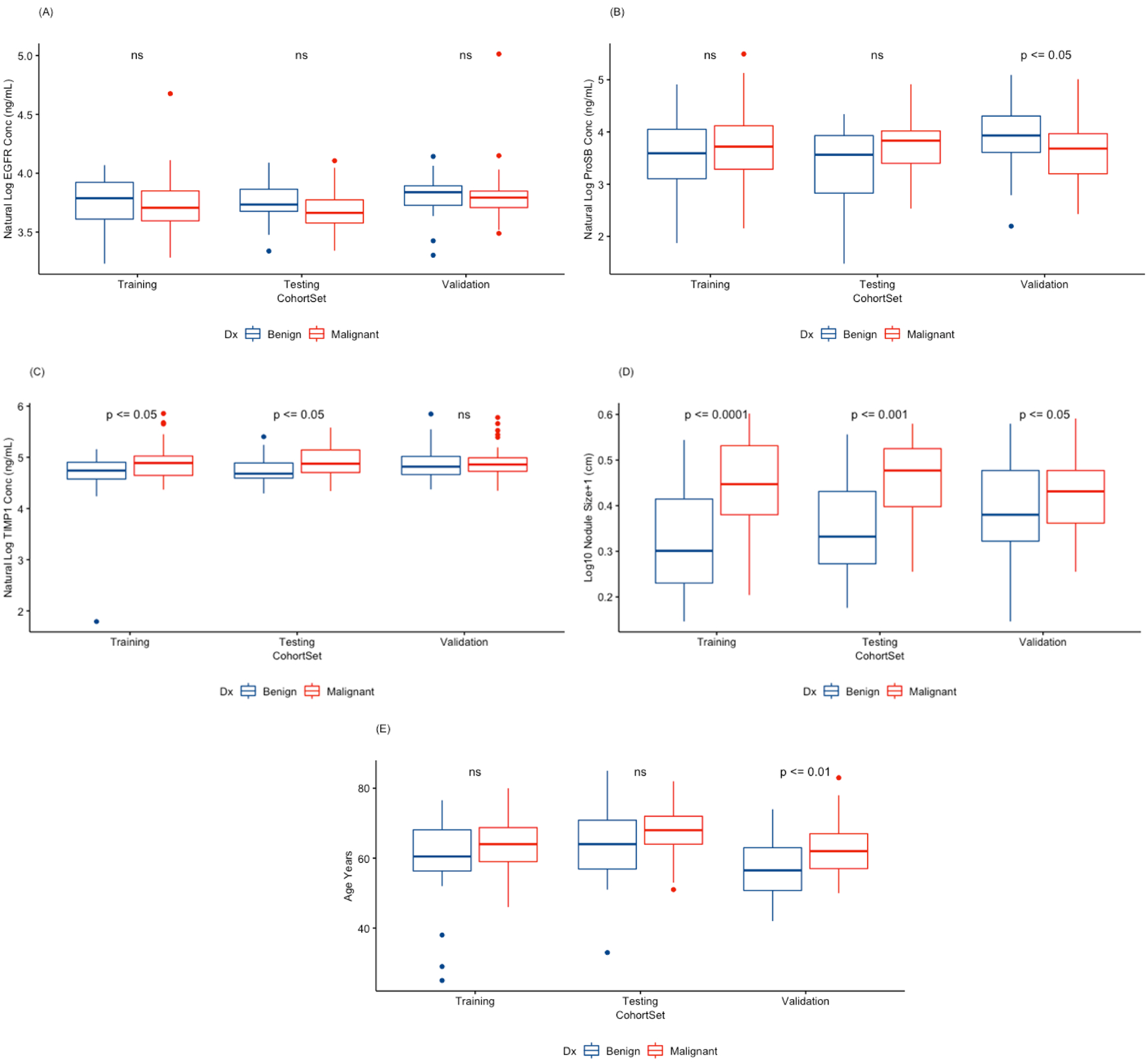
Distributions of protein biomarkers and clinical factors between DX (diagnosis) of benign (blue boxes) and malignant (red boxes) disease subjects by cohort set. Natural log of the (A) EGFR (Epidermal Growth Factor Receptor), (B) ProSB (Pro-Surfactant Protein B), and (C) TIMP1 (Tissue Inhibitor of Metalloproteinases 1) plasma levels, (D) logarithm base 10 of the lung nodule diameter +1 cm, and (E) subject age. The Training cohort set consists of 2/3 of the 8 medical center combined subjects. The Testing cohort set consists of the remaining 1/3 of the 8 medical center combined subjects. The Validation cohort set are the subjects from Vanderbilt University Medical Center. Statistically significant differences between the diagnoses within a cohort set are indicated by the p-value (above the pair of boxes). Not-significant (ns) were *p*-values ≥ 0.05.

Individual biomarker and clinical factor distributions and significance between benign and malignant nodule diagnoses are shown in Figure 2. The ranges of the variables are not dramatically different although significance varies between the sets for the biomarkers ProSB (Figure 2B) and TIMP1 (Figure 2C), and the clinical factor age (Figure 2E). Nodule size (Figure 2D) is significantly different between the benign and malignant nodules in the training and test sets, and much less so in the validation set.

### Algorithm performance in training, test, and validation sets

**Figure 3.**
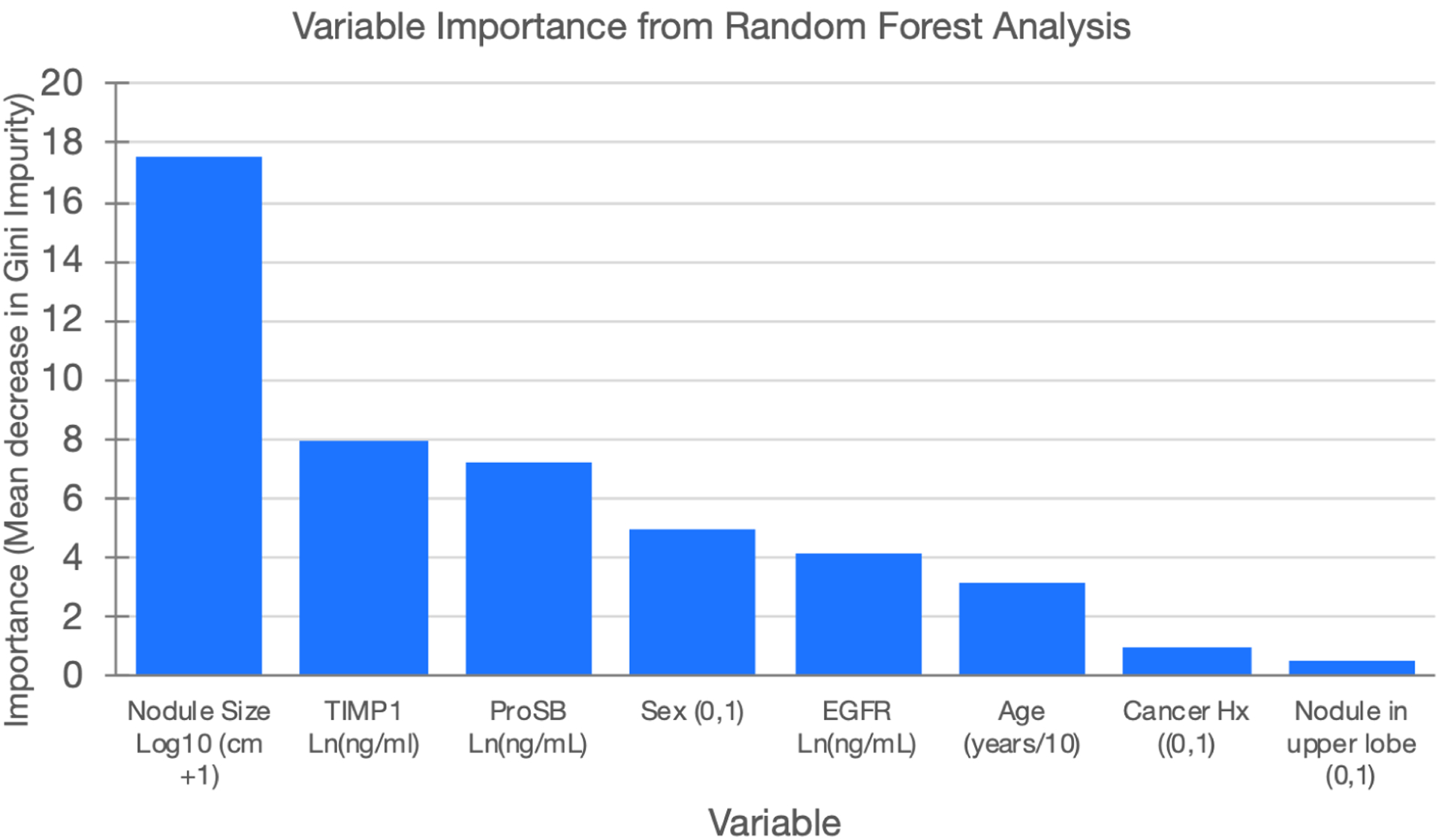
Variable importance in modeling the likelihood that a lung nodule is malignant based on the mean decrease in the Gini Impurity value from a random forest analysis. Nodule size is the logarithm base 10 of the lung nodule diameter +1 cm. TIMP1 (Tissue Inhibitor of Metalloproteinases 1), ProSB (Pro-Surfactant Protein B), and EGFR (Epidermal Growth Factor Receptor) are the natural log transformed plasma levels. Sex is the subject’s sex coded as 0=female and 1=male. Age is the subject age divided by 10. Cancer Hx is the subject’s history of cancer other than lung cancer. Location of the nodule in the upper lobe of either lung is coded as 0 = no and 1 = yes.

Variable selection of the specific 3 proteins and 3 clinical factors for the machine learning modeling was based on their relative importance in random forest analyses with the training set as shown in Figure 3. Nodule size was the most informative followed by TIMP1 and ProSB levels. Sex, EGFR, and subject age were also informative, while cancer history and nodule location were least informative and not included in the parsimonious model.

**Figure 4.**
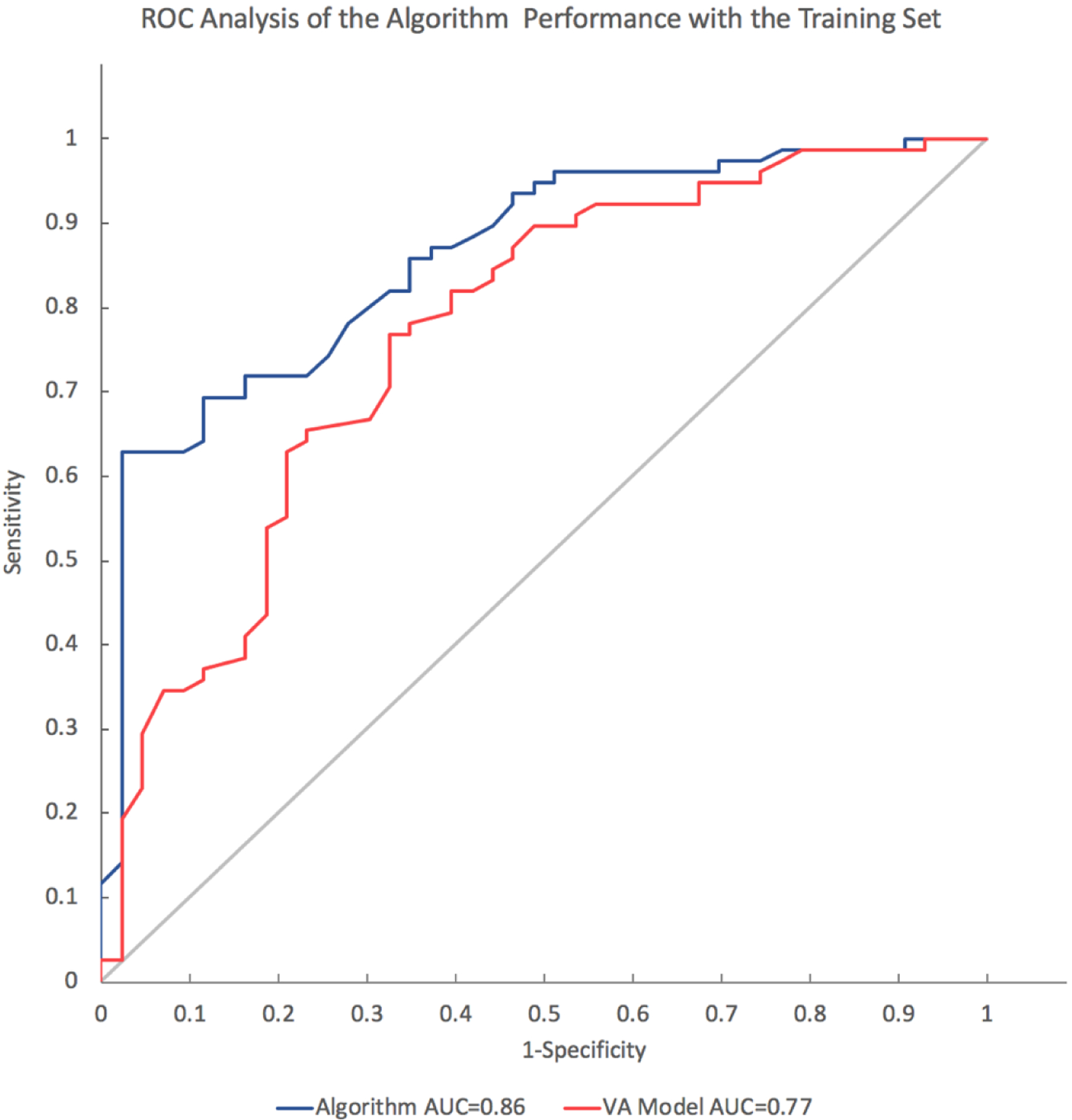
Receiver operator characteristic (ROC) plot of sensitivity vs. 1-specifcity for all cut-offs from 0 to 1 for the SVM model and the VA model in the training cohort set. The SVM algorithm ROC curve (blue line) has an area under the curve (AUC) of 0.86. The VA model ROC curve (red line) has an AUC of 0.77. The curve of no discrimination for reference is indicated by the gray diagonal line for which the AUC is 0.50.

The SVM training analysis identified an algorithm that provided optimal accuracy in nodule classification as well as nodule reclassification of those IPN classified as falling within the intermediate risk range by the VA model. The shape of the ROC curves for the SVM model with the training set shows higher specificity and sensitivity than the VA model. In the training set, the ROC area-under the curve (AUC) value for the SVM model was significantly higher (*p* = 0.006) at 0.86 (95% CI 0.79–0.93) than the VA Model at 0.77 (95% CI 0.68–0.86) (Figure 4). The validation set showed lower AUC values for both the VA model (0.70, 0.59 to 0.80) and the algorithm (0.64, 0.53 to 0.76) that were not significantly different (*p* = 0.2).

A score cutoff of 0.5 was identified from an analysis of the training data to maximize the overall accuracy as a tradeoff of sensitivity and specificity with an emphasis on higher sensitivity as a rule-out test. Using a single cut point of 0.5, with the training set prevalence of 64%, the algorithm NPV was 80% and the PPV was 78%. This compared to an NPV of 0% (no subject exhibited a pretest probability of malignancy < 0.05) and a PPV of 64% for the VA model using the published intermediate risk range cut points of 0.05 and 0.65.

In the validation set, the algorithm and the VA model showed NPV values of 84% and 0%, respectively using the 51% prevalence of disease in the validation set. With the 0.05 and 0.65 published risk range cut points for the VA model, only 30% of the samples were assigned a risk level (29.9% risk-predicted yield) compared to a 100% risk-predicted yield with the SVM model. With the 0.5 cutoff, the algorithm classified three subjects with malignant disease as falling below the cut point, resulting in a 94% sensitivity. Those three patients were men, 52–67 years old, with an IPN measuring 8 to 12 mm.

With a disease prevalence of 0.25% as expected in a typical community pulmonary practice [19–21], the predicted NPV of the SVM model would be 94% with a 32% PPV.

### Reclassification of indeterminate pulmonary nodules stratified by the VA Model with a pre-test probability within the intermediate risk range

Among the 97 validation set subjects, 68 where assigned by the VA Model as having a pre-test probability of malignancy falling within the intermediate risk range of 0.05 to 0.65. The algorithm correctly identified as benign or malignant 44 of those 68 (65%) indeterminate IPN. More specifically, 28 (41%) subjects with malignant nodules were correctly found to be true positive, while 16 (24%) with benign disease were appropriately identified as true negative. The net reclassification index was 29% as the sum of the correctly classified subjects (18 + 28 + 16) minus the sum of incorrectly identified subjects (11 + 3 + 21). Twenty-one subjects were classified as false positive. A sub-analysis revealed 11 were men and 10 were female, and their ages ranged from 50–74 years. Only two of these patients had nodules less than 10 mm; the remaining 19 subjects had an average IPN of 15 mm.

## Discussion

This evaluation is an important clinical validation study of the performance characteristics of a novel integrated proteomics test, comprising both proteins and clinical parameters, to accurately distinguish benign from malignant nodules in current smokers. As a large, multicenter, retrospective study, this work has several important findings. First, a ‘lower risk’ result accurately identifies patients with benign nodules to serve as a rule-out test. This approach may enable physicians to move from a nodule management strategy in which further testing is indicated to one in which serial surveillance is advisable. Thus, if incorporated into the current algorithm for managing nodules, this test may reduce a current smoker’s exposure to the morbidity and cost of avoidable invasive procedures. Second, very few malignancies are missed, and the clinical presentations of such patients indicates they are likely to be closely followed by their physician, thus increasing the chances the lung cancer will be identified on a subsequent clinical visit.

When choosing a strategy for evaluating patients with lung nodules, clinicians should consider both the probability that the nodule is malignant and the advantages and disadvantages of management strategies [19]. Serial surveillance has the advantage of being noninvasive and is recommended if the probability of cancer is less than 5% [19]. However, relatively few samples fall into this risk category. Despite the advances in imaging and nonsurgical biopsy techniques, invasive sampling of low-risk nodules and surgical resection of benign nodules remain common within community-based practices of pulmonologists [21]. At the other end of the spectrum, guidelines recommend nodules with a pretest probability of cancer greater than 65% be promptly resected in those healthy enough to tolerate surgery [19]. The harms associated with this strategy include a morbidity of 5% and a mortality of 0.5% [19].

Perhaps the most challenging group to manage are those with intermediate risk nodules that current guidelines define as a pretest probability of malignancy of 5% to 65%. Differentiating the minority of malignant from benign IPN represents one of the most urgent clinical problems in the early detection of lung cancer [22]. Although most indeterminate pulmonary nodules represent benign disease, significant morbidity and cost are associated with their management – up to $28 billion/year in the United States [22]. It is noteworthy that with the VA Model no subjects were identified as low risk. The proportions of indeterminate nodules falling into high, intermediate, or low risk for cancer vary with clinical setting, but the largest proportion (50–76%) falls win the intermediate-risk group [22]. Nodules in this group account for the largest number of invasive biopsies for benign disease [22].

Predicting the risk of developing lung cancer is a difficult task. Overdiagnosis is a serious problem in screening detected lung cancers [22]. Plasma protein biomarkers, such as the assay described herein, may be particularly important in helping to select those patients appropriate for serial surveillance by further enhancing the quantitative, noninvasive assessment of these nodules. When analyzing only those 68 IPN initially categorized by the VA Model as falling within the intermediate risk range, an additional 16 (24%) specimens with benign disease were appropriately identified as a true negative. At a NPV of 0.94, this may suggest the plasma protein assay may help to minimize harms of evaluating patients with benign disease. In this study, an additional 28 (41%) subjects with malignant nodules were correctly found to be truly positive, which may enable an earlier diagnosis of malignant nodules. Because 98% of the specimens in the independent validation set were Stage I and II lung cancers, those nodules may represent a potentially curable form of lung cancer when caught early enough to halt its progression. At a disease prevalence rate of 25%, and a sensitivity of 94%, the diagnostic yield (i.e., the number of individuals falling below the 0.5 cut point) of this test is 20%. Based on an assumed clinical decision for patients falling below the test cut point, it may be possible that 20% of unnecessary biopsies or surgeries could be avoided by using the test.

The performance of the SVM model in the validation cohort is lower, albeit not significantly lower than that in the training and test sets. The validation cohort was comprised of only 97 samples where even one mis-classification would have an impact on the AUC. Overall, the AUC confidence intervals in the independent validation set overlap those in the training set.

The current study has several limitations, including the need to more fully assess the test in other races, and how other conditions (such as obesity and its pro-inflammatory state, or steroid use) may affect the assay performance. This algorithm is also dependent on a compliant patient; those who do not adhere to follow-up appointments may have their cancer diagnosis missed. A clinical utility study to assess the impact of the algorithm on clinical decision making is also needed as outlined in the American Thoracic Society policy statement [23]. Ideally, long-term follow-up including the rate of lung cancer deaths prevented using this test is desired to verify this as an effective marker of aggressive lung cancer.

## Conclusion

Risk stratification for benign nodules is improved with the SVM model compared to current clinical practice methods. We hypothesize that patients with benign disease may benefit the most from this rule-out assay by avoiding unnecessary lung biopsy and subsequent overtreatment, while improving the quality of care and reducing the risk of harm from these procedures.

## Figures and Tables

**Table 1.** Subject set demographics. Diagnosis *p*-value refers to the significance of the difference in the characteristic between the Benign and Malignant diagnosis

Characteristic	Training Set	Testing Set	Independent Validation Set
Number of subjects, n	**121**	**59**	**97**
Age, mean (SD)[range], years	62.7 (9.1) [25–80]	65.9 (9.2) [33–85]	60.1 (8.4) [42–83]
Benign	60.8 (10.8) [25–76]	63.6 (10.6) [33–85]	57.3 (8.1) [42–74]
Malignant	63.8 (7.9) [46–80]	67.5 (7.8) [51–82]	62.8 (7.9) [50–83
Diagnosis P-value	0.117	0.133	< 0.001
**Sex, n (%)**			
Male	73 (60)	42 (71)	58 (60)
Benign	33 (45)	19 (45)	28 (48)
Malignant	40 (55)	23 (55)	30 (52)
Diagnosis P-value	0.321	0.513	0.853
Female	48 (40)	17 (29)	39 (40)
Benign	10 (21)	5 (29)	20 (51)
Malignant	38 (79)	12 (71)	19 (49)
Diagnosis P-value	< 0.001	0.04	
Pack-year, mean (SD)	49 (30)	45 (48)	60 (36)
Benign	44 (37)	48 (42)	57 (36)
Malignant	51 (26)	53 (34)	63 (37)
Diagnosis P-value	0.349	0.65	0.455
**Lung Nodule**			
Size, mean (SD)[range], mm	16.2 (7.1) [4–30]	16.4 (6.4) [5–28]	16.0 (6.0) [4–29]
Benign	11.7 (5.6) [4–25]	12.9 (5.6) [5–26]	14.9 (6.1) [4–28]
Malignant	18.7 (6.6) [6–30]	18.7 (5.8) [8–28]	17.2 (5.6) [8–29]
Diagnosis *p*-value	< 0.001	< 0.001	0.05
Location upper lobe, n (%)	64 (53)	38 (64)	55 (57)
Benign	17 (27)	14 (37)	27 (49)
Malignant	47 (73)	24 (63)	28 (51)
Diagnosis P-value	< 0.001	0.04	1
**Histology, n (%)**			
Benign nodule diagnosis	43 (36)	24 (41)	48 (49)
Granuloma	3 (7)	0 (0)	5 (10)
CT scan stable	12 (28)	8 (33)	41 (85)
Not reported	28 (65)	16 (67)	2 (4)
Malignant nodule type	78 (64)	35 (59)	49 (51)
Adenocarcinoma	18 (23)	13 (37)	28 (57)
Squamous cell	8 (10)	4 (11)	14 (29)
NSCLC	3 (4)	1 (3)	3 (6)
Other	0 (0)	0 (0)	4 (8)
Not reported	49 (63)	17 (49)	0 (0)
**Lung Cancer Stage, n (%)**			
Stage I & II	68 (87)	29 (83)	48 (98)
Stage III & IV	7 (9)	5 (14)	1 (2)
Not reported	3 (4)	1 (3)	0 (0)
**Race/Ethnicity, n (%)**			
White	96 (79)	51 (86)	91 (94)
Black	18 (15)	5 (8)	4 (4)
Asian	4 (3)	1 (2)	0 (0)
Hispanic	1 (1)	1 (2)	0 (0)
Other	2 (2)	0 (0)	2 (2)
Not reported	0 (0)	1 (2)	0 (0)

**Table 2.** SVM Model performance in the independent cohort used for validation.

Validation cohort; prevalence: 50.5%						
	SVM Model (single cutoff: 0.50)			VA Model (low cutoff: 0.05 and high cutoff: 0.65)		
Model Result, n	All	Malignant	Benign	All	Malignant	Benign
Total	97	49	48	97	49	48
High Risk	78	46	32	29	18	11
Intermediate Risk	0	0	0	68	31	37
Low Risk	19	3	16	0	0	0
Correct	62	46	16	18	18	0

Metric, %	Validation cohort (50.5% prevalence)	Predicted (25.0% prevalence)	Validation cohort (50.5% prevalence)	Predicted (25.0% prevalence)
PPV	59	31.9	62.1	10.9
NPV	84.2	94.2	0	0
Accuracy	63.9		18.6	
Sensitivity	93.9		36.7	
Specificity	33.3		0	
D_x_ Yield	19.6		0	
Risk-predicted Yield	100		29.9	

D_x_ Yield is the percent of all samples that are less than the lower cutoff i.e., [True Negative + False Negative]/Total number of samples. Risk-predicted Yield is the percent of all samples given a low-risk or high-risk result excluding those samples given an intermediate risk result. PPV=Positive Predictive Value defined as [sensitivity X prevalence]/[(sensitivity X prevalence) + (1-specificity) X (1-prevalence)]. NPV = Negative Predictive Value defined as [specificity X (1-prevalence)]/[(prevalence X (1-sensitivity)] + (specificity X (1-prevalence))].
